# The impact of mental toughness on subjective well-being among Chinese college students in the post-COVID-19 era

**DOI:** 10.3389/fpubh.2025.1556096

**Published:** 2025-07-07

**Authors:** Chunyue Zhu, Lin Peng, Shuo Li

**Affiliations:** ^1^School of Marxism, Chuzhou University, Chuzhou, China; ^2^School of Economics and Management, Chuzhou University, Chuzhou, China; ^3^School of Earth and Environment, Anhui University of Science and Technology, Huainan, China

**Keywords:** university students, depression, anxiety, personal resources, social support, subjective well-being, mental health

## Abstract

The COVID-19 pandemic has profoundly impacted mental health worldwide, particularly within campus environments, where it has heightened issues such as anxiety, depression, and stress. Despite the increasing recognition of these challenges, the role of psychological resilience—defined as personal and external support resources that aid in coping—remains underexplored in relation to mental health outcomes. To address this gap, the present study investigates the relationships between subjective well-being, psychological resilience, and mental health symptoms, measured using the DASS-21 (Depression, Anxiety, and Stress Scale), 3 years after the pandemic’s onset. In a cross-sectional survey of Chinese college students (*N* = 291), we utilized the DASS-21 and the Psychological Resilience Scale, along with multivariate linear regression modeling, to examine these relationships. The results indicated that both personal resilience factors and external support had significant effects on students’ subjective well-being. Specifically, emotional regulation, interpersonal support, and family backing had direct positive effects on well-being and indirectly enhanced happiness by reducing anxiety. Notably, goal orientation influenced well-being indirectly by moderating anxiety. Moreover, positive cognitive patterns exhibited a multifaceted impact on subjective well-being, affecting it through both direct and indirect pathways, particularly by reducing anxiety and depression. While anxiety played a central mediating role in these pathways, stress was also found to be a significant predictor of subjective well-being. These findings emphasize the critical importance of psychological resilience and underscore the multidimensional role of the DASS-21 in assessing university students’ mental health. The study offers a theoretical foundation for the development of sustainable mental health interventions tailored to the needs of students in the aftermath of global crises.

## 1 Introduction

The COVID-19 pandemic has had profound global repercussions, not only triggering a public health crisis but also exerting a significant impact on mental health (1). Social isolation, unemployment, and economic instability have contributed to widespread psychological issues, including heightened levels of anxiety, depression, and stress. The prevalence of anxiety disorders and depression has surged, leaving many individuals feeling increasingly helpless and despondent (2). These challenges are reflected in heightened fear and uncertainty about the future, accompanied by a general decline in quality of life (1). College students, in particular, have been severely affected, as they are at a critical juncture of managing academic pressures and preparing for future employment. This makes their mental health challenges especially pronounced (3). At this crucial stage of psychological development and social adaptation, the pandemic has intensified existing stressors, such as unemployment and limited social interaction, which may negatively impact their energy levels, focus, cognitive functioning, and optimism (4). These factors leave college students especially vulnerable to the adverse effects of the pandemic.

During the COVID-19 pandemic, the abrupt changes in lifestyle—including social isolation, the shift to online learning, and economic instability—have significantly increased the risk of mental health problems among university students (5). Among them, anxiety, depression and stress levels, as key indicators of negative emotions, can often evaluate the health problems faced by individuals (6). For example, elevated anxiety levels during the pandemic have been strongly associated with sleep disturbances (7) and academic burnout (8). Recent studies have reported a notable increase in anxiety, depression, and stress as a direct result of the pandemic, with these issues being particularly prevalent in the student population (9, 10). A study conducted among Chinese university students revealed that the prevalence of anxiety and depression reached 24.9 and 37.0%, respectively, during the early stages of the pandemic (11). Similar studies from other countries also highlight the significant rise in mental health problems among university students during this period (12, 13). Research further suggests that factors such as social isolation, the sudden shift to remote learning, and uncertainty about the future may have exacerbated these mental health challenges (14). The combination of these factors not only increases the likelihood of developing psychological disorders but also contributes to a negative overall climate, potentially impairing academic performance, social engagement, and, in severe cases, leading to self-harm (15). Therefore, it is crucial to explore the mental health status of university students during the pandemic, particularly focusing on uncovering the underlying mechanisms that impact their psychological well-being. This area of inquiry remains a key focus for contemporary psychological research.

Scholars have increasingly turned their attention to the potential interplay between negative emotions and psychological resilience in addressing pandemic-related mental health challenges (16, 17). The Depression, Anxiety, and Stress Scale (DASS-21) exhibits strong internal consistency and temporal stability, effectively differentiating between anxiety and depression. It serves as an excellent instrument for measuring psychological traits in both clinical and non-clinical settings (18). In a study conducted by Alkhamees et al. (19), the DASS-21 revealed that higher scores were associated with individuals in the workforce, women, students, and those with pre-existing mental health conditions. Psychological resilience, as a vital resource for promoting adaptive functioning, plays a crucial role in alleviating psychological distress in the face of significant threats (20). Previous research has demonstrated that resilience acts as a protective factor, reducing the negative effects of exposure to traumatic life events in high-stress environments (21). However, research exploring the combined effects of negative emotions and psychological resilience on university students’ mental health remains limited, particularly within the unique context of the COVID-19 pandemic.

Due to the broad scope of psychological resilience, its effectiveness in psychological interventions can sometimes be diluted. To address this limitation, the Psychological Resilience Scale classifies goal focus, emotional regulation, and positive cognition as personal resources, while family support and interpersonal assistance are categorized as supportive resources. This distinction facilitates the identification of key factors that contribute to adolescent recovery from adversity (22). In this study, personal resources, supportive resources, and negative emotions are integrated and examined using a multivariable linear regression model to quantify the relationships among subjective well-being, psychological resilience, and DASS-21 scores. In conjunction with a mediation analysis, this approach aims to explore how these variables influence university students’ subjective well-being and mental health. Specifically, the study seeks to: (1) analyze the comprehensive mechanisms by which personal resources (e.g., emotional regulation, goal focus, and positive cognition) and supportive resources (e.g., family support and interpersonal assistance) impact university students’ mental health; (2) investigate the mediating role of negative emotions in the relationship between psychological resilience and subjective well-being; (3) By revealing the mechanisms of these factors in special contexts, to provide a scientific basis and precise guidance for psychological intervention strategies in response to similar public health emergencies in the future, promoting the sustainable development of university students’ mental health.

## 2 Materials and methods

### 2.1 Study design and participant recruitment

This study employed a cross-sectional observational design, conducted between March and April 2024, utilizing the Chinese online survey platform “Wenjuanxing.” A snowball sampling method was applied to recruit full-time undergraduate students from Chuzhou University. Prior to participation, all respondents were fully informed about the study’s objectives and procedures, and their informed consent was obtained. A total of 293 questionnaires were collected, with two invalid responses excluded, resulting in a final sample size of 291 participants. The mean age of the respondents was 19.4 years, and 68.7% were female (*n* = 200). Additionally, 41.6% (*n* = 121) of the sample resided in urban areas, and 76% (*n* = 225) reported being in good physical health. To ensure participant privacy, personal identifiers such as student names could not be traced through ID numbers. The confidentiality of the data was rigorously upheld, with access restricted solely to members of the research team.

### 2.2 Subjective happiness measurement

The primary outcome of this study is Subjective Well-Being (SWB), which evaluates an individual’s overall perception of life quality based on their personal standards (23). The SWB scale includes 15 items, each scored on a 5-point Likert scale. The total score is calculated by summing the item responses, with higher scores indicating greater levels of well-being. SWB levels are divided into three categories based on standard deviations: scores between 0 and 42.243 indicate low well-being, scores between 42.243 and 54.857 represent moderate well-being, and scores between 54.857 and 60 indicate high well-being.

### 2.3 Psychopathological assessment tools

This study employs the DASS-21 to evaluate the tripartite model of psychopathology, serving as a comprehensive measure of overall distress (24). The DASS-21 comprises 15 items, which are categorized into three subscales: Depression, Anxiety, and Stress. The Depression subscale assesses symptoms such as irritability, hopelessness, life devaluation, self-deprecation, lack of interest or involvement, anhedonia, and inertia. The Anxiety subscale measures autonomic arousal, skeletal muscle tension, situational anxiety, and the subjective experience of anxious feelings. The Stress subscale evaluates levels of chronic nonspecific arousal, including difficulty relaxing, nervous excitement, ease of becoming upset or agitated, irritability or overreaction, and impatience. Each item is scored according to the 4-level Likert scale. The higher the score, the lower the severity of depression, anxiety and stress symptoms.

### 2.4 Resilience level assessment

Resilience levels were evaluated using the Psychological Resilience Scale (PRS) (25), a tool comprising 20 items scored on a 5-point Likert scale. The scale assesses five key dimensions: goal orientation, emotional regulation, positive cognition, family support, and interpersonal assistance.

### 2.5 Data analysis methods

Descriptive statistics were conducted to summarize the sociodemographic and clinical characteristics of the sample across the three levels of subjective well-being.

Construct a multivariate linear regression model to test the role of negative emotions and psychological resilience as predictors of subjective well-being (main results evaluated using the Subjective Well Being assessment) (26). In the study, the model adjusted for demographic characteristics such as age, gender, and health status and converted them into factor variables. During the research period, all variables were managed.

A hierarchical multiple regression model was employed to assess the incremental effect of goal focus on predicting behavioral outcomes (component scores) (27). In the first step, demographic variables (gender, age, and place of residence) were entered as predictors. In the second step, depression, anxiety, and stress levels from the DASS-21 were added to the model. Finally, five dimensions of psychological resilience Scale, including goal concentration, emotional control and positive cognition, are added to the model. Additionally, a mediation model using SPSS version 25 was applied to explore how negative emotions, personal resilience, and external support interact to influence subjective well-being. All missing data have been processed using multiple imputation methods. For all analyses, the statistical significance level is set to *p* < 0.05.

## 3 Results

### 3.1 Depression, stress, and anxiety shape an individual’s experience of subjective well-being

Through the analysis of a multivariate linear regression model, we found that depression, stress, and anxiety significantly impact an individual’s subjective well-being. Notably, anxiety emerged as a significant negative predictor, with an estimated coefficient of 0.563 (*p* = 0.009), indicating a substantial negative effect on well-being (Table 1). This finding was corroborated by the hierarchical linear regression model, which reported estimated coefficient for anxiety of 0.589 (**p = 0.006), demonstrating both consistency across models and an even stronger negative impact (Table 2).

**Table 1:** Multivariate linear regression model.

Variable	Estimate	Std. error	*t*-value	Pr(>|t|)
Intercept	12.521	6.509	1.924	0.055
Region-Rural	−0.288	0.651	−0.443	0.658
Health Level-Health	0.296	0.778	0.381	0.704
Health Level-Poor	−5.429	5.440	−0.998	0.319
DASS-21-Pressure	0.028	0.207	0.137	0.891
DASS-21-Anxiety	0.563	0.215	2.621	0.009**
DASS-21-Depression	0.065	0.172	0.379	0.705
Age	0.755	0.304	2.483	0.014*
Gendermale	1.002	0.678	1.478	0.141
PRS-Target Focus	0.033	0.125	0.265	0.791
PRS-Emotional Control	0.648	0.159	4.077	<0.001***
PRS-Positive Cognition	0.056	0.123	0.457	0.648
PRS-Family Cognition	0.277	0.183	1.512	0.132
PRS-Interpersonal Assistance	0.359	0.155	2.319	0.021*

***p < 0.001; **p < 0.01; *p < 0.05.

**Table 2:** Hierarchical linear regression model.

Variable	Estimate	Std. error	*t*-value	Pr(>|t|)
Intercept	49.251	1.944	25.337	<0.001***
Gender	−1.686	0.785	−2.149	0.032*
Age	0.008	0.036	0.225	0.822
Health	1.585	0.843	1.881	0.061
Intercept	41.892	2.034	20.593	<0.001***
Gender	−1.129	0.726	−1.556	0.121
Age	0.012	0.033	0.367	0.714
Health	−0.012	0.800	−0.015	0.988
DASS-21-Depression	0.250	0.168	1.487	0.138
DASS-21-Anxiety	0.601	0.228	2.635	0.009**
DASS-21-Pressure	0.243	0.218	1.113	0.267
Intercept	29.472	3.079	9.572	<0.001***
Gender	−0.879	0.675	−1.302	0.194
Age	0.012	0.031	0.398	0.691
Health	−0.563	0.749	−0.751	0.453
DASS-21-Depression	0.114	0.172	0.664	0.507
DASS-21-Anxiety	0.589	0.214	2.754	0.006**
DASS-21-Pressure	−0.044	0.206	−0.212	0.832
PRS-Target Focus	0.039	0.126	0.310	0.756
PRS-Emotional Control	0.626	0.159	3.936	<0.001***
PRS-Positive Cognition	0.047	0.122	0.384	0.701
PRS-Family Cognition	0.309	0.184	1.683	0.093
PRS-Interpersonal Assistance	0.351	0.155	2.263	0.024*

***p < 0.001; **p < 0.01; *p < 0.05.

Stress was not a significant predictor in the multivariate linear regression model (*p* = 0.891), with an estimated coefficient of 0.028. Similarly, in the hierarchical linear regression model, the coefficient was −0.044 (*p* = 0.832), suggesting a potential negative effect on well-being, although it remained non-significant in both models. This lack of significance may be attributed to other factors, such as emotional regulation or anxiety, playing a more dominant role in the models, thereby diminishing the direct effect of stress.

### 3.2 The role of personal resources in enhancing subjective well-being

**Figure 1:**
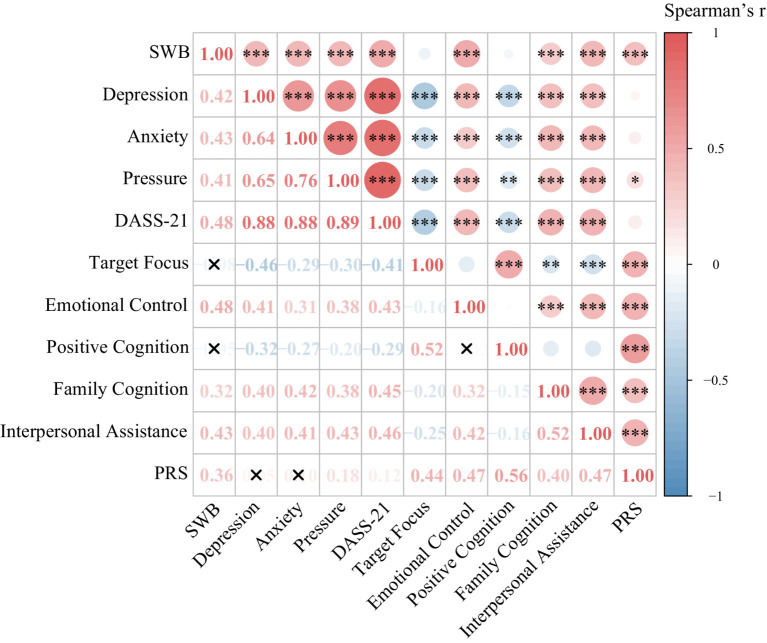
Research on the correlation between the dimensions in the standardized scale. The degree of color ladder indicates the intensity of bivariate correlation, where the cool color (blue series) represents the positive correlation and the warm color (red series) indicates the negative correlation. The horizontal and vertical coordinates represent the dimensions of the scale. Statistical significance: ***p < 0.001; **p < 0.01; *p < 0.05 (two-tail test).

The results from both the multivariate and hierarchical linear regression models demonstrate that emotional regulation is a significant factor in enhancing subjective well-being, with estimated coefficients of 0.648 and 0.626, respectively, and *p*-values of less than 0.001 (Tables 1, 2). Additionally, correlation analysis revealed a positive correlation between emotional regulation and subjective well-being, with a correlation coefficient of 0.48 (***p < 0.001) (Figure 1). These findings underscore the critical role of emotional regulation in improving subjective well-being.

Although positive cognition and family support were significant, their effects on subjective well-being did not reach statistical significance (positive cognition: *B* = 0.056, *p* = 0.648; family support: *B* = 0.277, *p* = 0.132) (Table 1). This may indicate that, within the sample of this study, the influence of positive cognition and family support was relatively weak or possibly overshadowed by other more prominent psychological factors.

Notably, interpersonal support, as a component of social support, had a significant positive effect on subjective well-being in the multivariate linear regression model (Table 1; *B* = 0.359, *p = 0.021). The correlation analysis further revealed a positive association between interpersonal support and individual well-being, with a correlation coefficient of 0.43 (Figure 1; ***p < 0.001).

### 3.3 The mediating effects of psychological stress, personal resources and social support on well-being

Through the construction of a mediation model, we analyzed the combined influence of psychological stress, personal resources, and social support on subjective well-being. In the mediation analysis between emotional regulation and stress, the direct effect of emotional regulation on subjective well-being was 0.8791 (***p < 0.001). Furthermore, the indirect effect of emotional regulation via anxiety was 0.2303, which was also statistically significant (Figure 1).

Conversely, the indirect effect of goal focus on subjective well-being through anxiety was negative and significant, with an effect size of −0.106 and a confidence interval of [−0.2220, −0.0178] (Table 3). The indirect effects of goal focus via depression and stress were −0.1334 and −0.0354, respectively, with confidence intervals of [−0.0478, 0.4130] and [−0.1207, 0.0398], indicating that the influence of goal focus on subjective well-being through depression and stress was not significant (Table 3). Moreover, the direct effect of goal focus on well-being was *B* = 0.183, suggesting that its direct impact was also not significant (Table 3).

**Table 3:** Mediation effect model.

Independent variables	Direct effects		Indirect effect					
	Effect sizes	*p*-value	Depression	Confidence interval	Anxiety	Confidence interval	Stress	Confidence interval
Target focus	0.183	0.120	−0.133	[−0.0478, 0.4130]	−0.106	[−0.2220, −0.0178]	−0.035	[−0.1207, 0.0398]
Emotional control	0.879	<0.000***	0.052	[−0.0640, 0.1973]	0.230	[0.0756, 0.4146]	−0.006	[−0.1756, 0.1621]
Positive cognition	0.210	0.067	−0.093	[−0.2013, −0.0067]	−0.116	[−0.2369, −0.0259]	−0.030	[−0.1152, 0.0381]
Positive cognition	0.753	<0.000***	0.083	[−0.0966, 0.2542]	0.244	[0.0396, 0.5073]	0.074	[−0.1032, 0.2686]
Interpersonal assistance	0.699	<0.000***	0.073	[−0.0510, 0.1933]	0.199	[0.0483, 0.3825]	0.018	[−0.1418, 0.1762]

***p < 0.001; **p < 0.01; *p < 0.05.

In the analysis of health status, the results indicated that the coefficient for “good health” was positive but not statistically significant (Table 1; *B* = 0.296, *p* = 0.704), while the coefficient for “poor health” was negative but also non-significant (Table 1; *B* = −5.429, *p* = 0.319), suggesting that health status had a relatively minor impact on well-being. However, interpersonal support demonstrated a significant positive effect on well-being (Table 1; *B* = 0.359, *p = 0.021).

Additionally, the effects of residence and gender on subjective well-being were not significant, indicating that these demographic factors have a relatively minor influence on well-being. In contrast, age exhibited a significant positive effect on subjective well-being (Table 1; *B* = 0.755, *p = 0.014), suggesting that well-being tends to increase with age. This may be attributed to older individuals having accumulated greater life experience and developed more effective coping strategies when dealing with challenges.

## 4 Discussion

### 4.1 The role of personal resource factors in enhancing subjective well-being

In the Post-COVID-19 Era, college students’ ability to regulate their emotions significantly impacted their subjective well-being. Emotional control not only directly enhanced students’ well-being but also indirectly improved their happiness by mitigating the effects of negative emotions. This finding is consistent with previous literature, which highlights emotional regulation as a strategy that helps individuals effectively enhance subjective well-being and life satisfaction (28). This mechanism has been supported by existing research, such as Gross’s emotion regulation theory, which underscores the pivotal role of emotional control in mental health (29). The present study demonstrated that the direct effect of emotional control on subjective well-being was particularly pronounced (Table 2; β = 0.626, *p* < 0.001), with a significant positive correlation between subjective well-being and emotional control (Figure 1; *r* = 0.48, *p* < 0.001). Thus, during crises such as the pandemic, emotional control can significantly and directly enhance well-being. Moreover, by regulating their emotions, college students were better able to cope with the negative emotions brought on by the pandemic, maintaining higher levels of life satisfaction and happiness. Anxiety, in particular, had a detrimental effect on subjective well-being during the pandemic, significantly diminishing it (30). However, students with stronger emotional regulation abilities were more likely to sustain a positive psychological state despite pandemic-related anxiety, thereby boosting their well-being (31). This study also found that emotional control had a significant indirect effect on subjective well-being by reducing anxiety levels, as measured by the DASS-21 (Table 3). This regulatory mechanism highlights the crucial role of emotional control in psychological health interventions, especially during unforeseen public health crises. Overall, emotional control profoundly influences college students’ subjective well-being through both direct and indirect pathways. Future psychological interventions should focus on enhancing students’ emotional regulation skills to better equip them for coping with sudden challenges like the COVID-19 pandemic, thereby improving their overall well-being.

This study found that while college students’ goal focus ability does not have a significant direct effect on subjective well-being, it underscores the importance of indirect pathways. Specifically, goal focus indirectly enhances subjective well-being by influencing negative emotions, such as reducing anxiety (Table 3). Research indicates that negative emotions significantly impact mental health, especially under the stress of unexpected events (32). For instance, adolescents who set clear goals are better able to manage their psychological state, engage in activities for extended periods, and potentially improve clinical outcomes (33). Additionally, a strong goal focus provides actionable guidelines, which can mitigate anxiety related to procrastination (34). Thus, self-set goals may help students avoid the repetitive rumination of negative emotions caused by the pandemic by focusing on controllable tasks and outcomes, thereby alleviating anxiety. Overall, while anxiety during the pandemic directly reflects emotional distress and psychological pressure among college students, goal focus can indirectly enhance subjective well-being by reducing anxiety. This finding highlights the need for future intervention strategies to concentrate on developing goal-setting skills to help college students better manage sudden events like the pandemic, thereby improving their well-being and mental health.

It is noteworthy that the direct effect of positive cognition on subjective well-being approaches significance (Table 3; *p* = 0.067), suggesting that the results did not meet the conventional threshold for statistical significance. However, existing literature indicates that positive thinking can enhance life satisfaction (35). Moreover, when assessing psychopathological states, negative thinking often outweighs positive thinking (36). Consequently, the complexity of emotional responses in high-pressure situations like a pandemic may cause the direct effect of positive cognition not to reach statistical significance. Positive cognition might also influence subjective well-being through a more complex mechanism involving the regulation of negative emotions such as anxiety and depression. Specifically, in this study, positive cognition indirectly improved subjective well-being by reducing anxiety and depression. This is because, in dealing with the uncertainties triggered by the COVID-19 pandemic, positive cognition helps individuals regulate their emotions more effectively and mitigate negative psychological responses. Additionally, Fredrickson emphasized that through cognitive broadening, positive emotions can spiral, which in turn enhances emotional well-being (37). However, stress, a common reaction to unexpected events, did not show a significant effect on well-being in this study. This may be due to the adaptive coping mechanisms employed by most students during the pandemic. There is a significant correlation between stress and coping mechanisms (38). Therefore, unlike the purely negative effects of anxiety and depression, moderate stress may sometimes improve performance. Overall, positive cognition has a multi-layered impact on subjective well-being through direct and indirect pathways, with the indirect effect through reducing anxiety and depression being more prominent. It is recommended that mental health education for college students emphasize cognitive restructuring and positive thinking training to effectively mitigate the impact of negative emotions and bolster psychological resilience.

### 4.2 The impact of support on subjective well-being

The study found that interpersonal assistance has a significant direct effect on subjective well-being (Table 3; β = 0.699, *p* < 0.001). This finding aligns with the research by Taniguchi (39). Additionally, McBeath et al. emphasized that students’ sense of belonging to their peers and access to high-quality peer support are closely linked to their overall mental health and well-being (40). On university campuses, which facilitate interactions among student groups, interpersonal support is particularly effective, representing a unique advantage for college students. However, this advantage has been greatly limited by the pandemic’s stay-at-home lockdown, which can lead to a sense of isolation and a lack of belonging (41), which significantly decreased their subjective well-being. Therefore, enhancing online communication and remote support to help students maintain effective social connections in a confined environment could be a crucial strategy for improving their mental health and well-being.

The study results indicate that family support has a significant and strong direct effect on subjective well-being (Table 3; β = 0.753, *p* < 0.001). This is mainly due to the fact that family environmental factors have had a significant impact on psychological distress during the pandemic. However, adolescents may experience varying interaction patterns when spending extended periods with family members (42). Specifically, there is a mechanism of action among family members, which can make the perceived fear of family members deepen their own fear, potentially worsening mental health issues (42). Conversely, healthy family interactions can alleviate psychological stress and promote relaxation, thus reducing pandemic-related psychological symptoms (43). Additionally, increased family life satisfaction can effectively reduce feelings of loneliness and enhance happiness (44). During the pandemic, as college students spent more time in home isolation with their families, the heightened interaction significantly amplified the impact of family factors on their well-being.

Additionally, interpersonal assistance and family support not only have direct effects on subjective well-being but also indirectly influence well-being by affecting negative emotions. Social support enhances individuals’ subjective well-being by alleviating anxiety. The study results indicate that when anxiety is considered as a mediator, it is significantly influenced by all dimensions of personal resources and social support (Table 3). This may be because anxiety is often associated with uncertainty in the social environment (45), whereas depression and stress are more related to internal emotional management. Particularly in the context of the pandemic, this uncertainty has been exacerbated (46), making anxiety more susceptible to influence. Therefore, compared to depression and stress, anxiety is more directly impacted by psychological resilience. Overall, anxiety plays a central role in the relationship between psychological resilience and subjective well-being. In designing psychological intervention strategies, prioritizing anxiety and enhancing family support and interpersonal assistance can significantly improve individuals’ subjective well-being.

## 5 Limitations of the study and future research directions

There are some limitations to this study. First, using cross-sectional design can only collect data at the same point in time, making it difficult to determine the reasons behind causation. Therefore, future studies should consider longitudinal designs to better capture the dynamic changes between variables and their causal paths over time. Second, the sample is limited to university students from Chinese institutions, so validating the findings in broader cultural and regional contexts is recommended to enhance the generalizability of the results. Additionally, this study did not account for other potential variables, such as self-esteem and socio-economic status. Future research should explore the moderating or mediating roles of these variables to gain a more comprehensive understanding of the mechanisms underlying students’ mental health.

**Figure 2:**
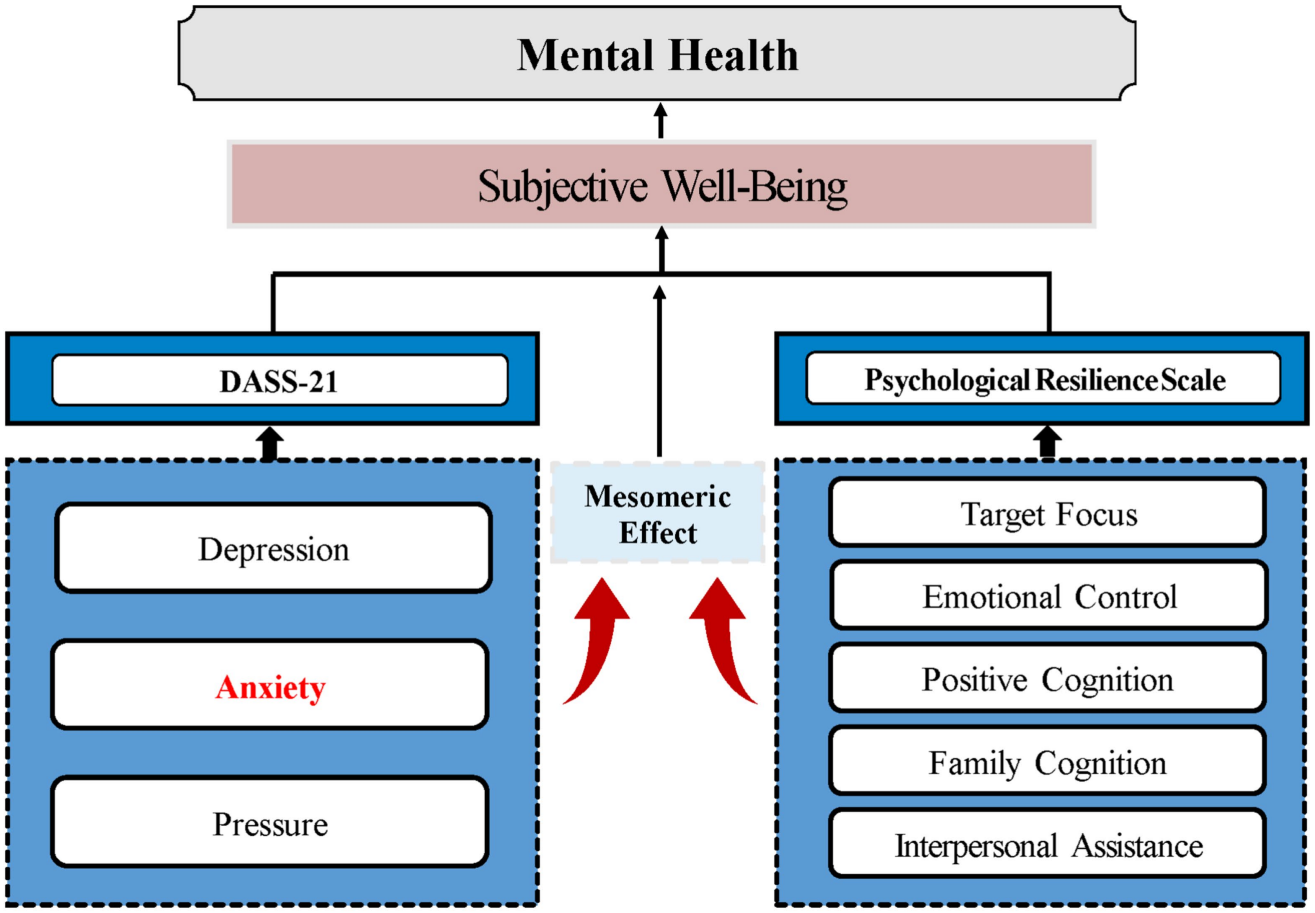
Conceptual diagram.

The analysis reveals that both negative emotions and psychological resilience can serve as mediators affecting subjective well-being, with potential complex interactions between the two (Figure 2). For example, interpersonal assistance and family support may indirectly enhance well-being by reducing anxiety, and a decrease in anxiety could subsequently improve an individual’s ability to benefit from social support. This suggests a complex interaction model. Future research should further investigate the mechanisms underlying these interactions to better understand how to enhance subjective well-being in college students through the modulation of social support and emotional states. Based on the findings of this study, it is suggested that education departments and universities should formulate systematic intervention strategies from the following dimensions: (1) Integrate peer support networks with professional psychological counseling services to develop tiered intervention programs tailored to students at varying risk levels; (2) Cognitive reappraisal, goal-oriented training, and other evidence-based techniques are integrated into mandatory mental health courses to enhance skill transfer effectiveness via scenario simulation and digital twin technology; (3) Restructure the design of public Spaces to facilitate informal social interactions, while developing an AI emotional companionship platform to seamlessly connect offline to online support networks.

## Data Availability

The original contributions presented in the study are included in the article/supplementary material. Further inquiries can be directed to the corresponding author.
